# Diagnostic and prognostic values of endothelial-cell-specific molecule-1 with malignant pleural effusions in patients with non-small cell lung cancer

**DOI:** 10.18632/oncotarget.17455

**Published:** 2017-04-27

**Authors:** Guo-Jun Lu, Cheng-Jie Shao, Yu Zhang, Yong-Yue Wei, Wei-Ping Xie, Hui Kong

**Affiliations:** ^1^ Department of Respiratory and Critical Care Medicine, The First Affiliated Hospital of Nanjing Medical University, Nanjing 210029, China; ^2^ Department of Respiratory Medicine, Nanjing Chest Hospital, School of Medicine, Southeast University, Nanjing 210029, China; ^3^ Department of Biostatistics, School of Public Health, Nanjing Medical University, Nanjing 210029, China

**Keywords:** ESM-1, non-small cell lung cancer, overall survival (OS), prognosis, biomarker

## Abstract

Over-expressed endothelial-cell-specific molecule-1 (ESM-1) in tumor vascular endothelium contributes to tumor angiogenesis, metastasis, and poor prognosis. However, the content of ESM-1 in pleural effusion is unclear. A retrospective study was carried out to investigate the diagnostic and prognostic values of ESM-1 with malignant pleural effusions in patients with non-small cell lung cancer (NSCLC). ESM-1 levels in malignant pleural effusion (MPE) from 70 patients with NSCLC and 50 cases of benign pleural effusion (BPE) were measured using enzyme-linked immunosorbent assay. Receiver operating characteristic (ROC) curve was calculated to assess the diagnostic value of ESM-1. Survival curves were performed by Kaplan-Meier method and survival characteristics were compared by log-rank test. Univariable and multivariate Cox proportional hazards model were carried out to analysis the significance of different prognostic factors for overall survival (OS). ESM-1 levels were significantly higher in MPE than those in BPE (p < 0.001). By ROC curve analysis, with a cutoff level of 19.58 ng/ml, the accuracy, sensitivity, and specificity for ESM-1 diagnosis MPE were 82.5%, 81.4%, and 84.0%, respectively. Moreover, NSCLC patients with pleural fluid ESM-1 levels below 19.58 ng/ml had significant longer OS than those patients with higher levels (22.09 months vs. 11.49 months, p = 0.003). Multivariate survival analysis showed that high MPE ESM-1 level was an independent prognostic factor (HR, 1.007; p = 0.039) for the OS of NSCLC patients. This study showed that ESM-1 level in pleural effusion could be a potential diagnostic and prognostic marker in NSCLC patients with MPE.

## INTRODUCTION

Lung cancer is the leading cause of cancer-related death in the world [1], and non-small cell lung cancer (NSCLC) accounts for 85% of lung cancer cases [2]. Malignant pleural effusion (MPE) is a common complication in NSCLC patients and an independent factor for poor survival [3]. Since the treatment and prognosis are different, it is important to distinguish MPE from benign pleural effusion (BPE) to plan appropriate management. Till now, several approaches including thoracocentesis, closed pleural biopsy, and thoracoscopy have been used to obtain the pleural effusion for the cytologic or histologic diagnosis. Additionally, if cytology is negative, tumor biomarkers in pleural effusion such as carcinoembryonic antigen (CEA), cytokeratin 19 fragments (CYFRA 21-1) and carbohydrate antigen 153 (CA153) can be used for the identification of those patients with a high clinical suspicion for malignancy [4–6]. However, the low accuracy of these biomarkers limits their clinical diagnostic value [7]. Therefore, it is essential to search more accurate non-invasive biomarkers for the differential diagnosis of pleural effusion.

Endothelial-cell-specific molecule 1 (ESM-1), a 50-kD soluble dermatan sulfate proteoglycan specifically secreted by vascular endothelial cells, was initially cloned in 1996 by Lassalle and colleagues [8]. ESM-1 can be up-regulated by inflammatory cytokines and proangiogenic growth factors [9]. Previous studies indicated that ESM-1 was over-expressed in various malignant tumors tissue including NSCLC [9–14]. In gastric carcinoma and colorectal cancer, high serum levels of ESM-1 can be served as a marker for diagnosis and poor prognosis [15–17]. Moreover, ESM-1 mRNA is also over-expressed in NSCLC and high levels of circulating ESM-1 seem to be a predictor of poor prognosis in NSCLC [9]. However, the level of ESM-1 in NSCLC patients with MPE remains unclear and its role in the diagnosis and prognosis of NSCLC has not been investigated.

In the present study, we assessed levels of ESM-1 in MPE from NSCLC patients by using enzyme-linked immunosorbent assay (ELISA) and explored its diagnostic and prognostic value.

## RESULTS

### ESM-1 level in pleural effusion

**Figure 1:**
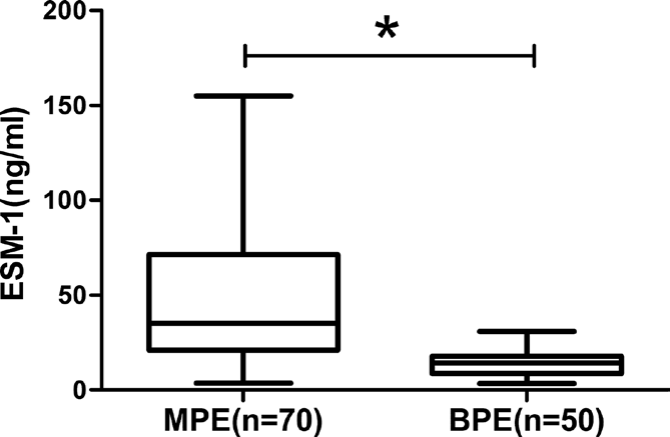
The expression of ESM-1 in pleural effusion MPE presents higher ESM-1 concentration than those with BPE (*p < 0.001).

A total of 120 eligible patients were included, with 70 of MPE and 50 of BPE. The baseline characteristics of NSCLC patients with MPE and patients with BPE are summarized in Table 1 (Table 1). No significant differences were found between MPE and BPE groups in the distribution of age and gender. As shown in Figure 1, the ESM-1 concentration in MPE from NSCLC patients was significantly higher than those in BPE (51.17 ± 4.51 ng/ml vs. 13.67 ± 0.92ng/ml, *p* <0.001) (Figure 1).

**Table 1:** The baseline characteristics of patients

Variable	MPE	BPE	*P*-value
Patients	70	50	
Mean age, years	59.34 ± 1.56	56.60 ± 2.31	0.176
Gender			0.225
Male	46 (65.71%)	38 (76%)	
Female	24 (34.29%)	12 (24%)	
Smoking condition			
Smoker	34 (48.57%)	29 (58.00%)	0.308
Nonsmoker	36 (51.43%)	21 (42.00%)	
BPE			
Tuberculous	ND	40	
Parapneumonic	ND	10	
Diagnostic method			
Biochemistry	ND	42	
Cytology	65	ND	
Pleural biopsy	5	8	

MPE = malignant pleural effusion; BPE = benign pleural effusion; ND = no data.

### Diagnostic value of ESM-1 in MPE

**Figure 2:**
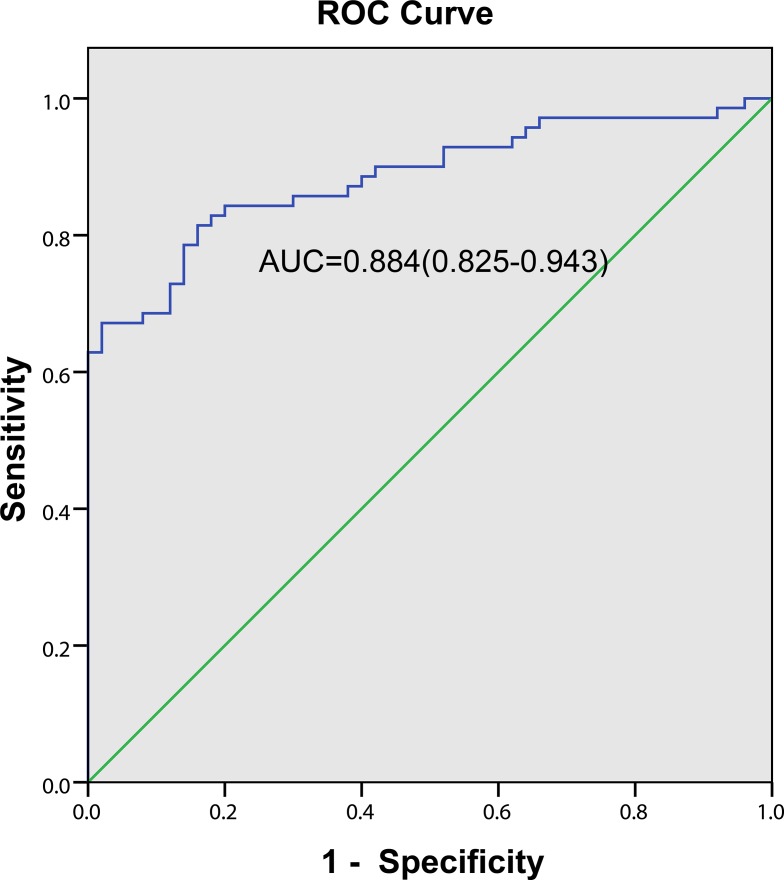
Diagnostic value of pleural fluid ESM-1 level for NSCLC patients with MPE The ROC curve discrimination of MPE and BPE according to pleural fluid ESM-1 concentrations (AUC = 0.884; 95% CI = 0.825–0.943) with a cutoff value of 19.58 ng/ml (sensitivity 81.4%; specificity 84.0%).

Based on the ROC curve, the diagnostic threshold of ESM-1for MPE was >19.58 ng/ml (Figure 2) with the area under the curve (AUC) was 0.884 (95% CI 0.825–0.943). Using a cutoff point of 19.58 ng/ml, ESM-1 had a sensitivity of 81.4% (57 of 70 patients), a specificity of 84.0% (42 of 50 patients), an accuracy of 82.5% (99 of 120 patients), a positive predictive value of 87.7% (57 of 65 patients) and a negative predictive value of 76.4% (42 of 55 patients).

### Relationship between ESM-1 concentration and clinicopathological factors in NSCLC patients with MPE

The association between ESM-1 concentration of MPE and clinicopathological factors in NSCLC patients was summarized in Table 2 (Table 2). In advanced NSCLC patients, ESM-1 concentration in MPE was associated with distant metastasis. Patients diagnosed with stage M1b (with metastasis to brain, hepatic or bone) had higher level of ESM-1 in MPE (*vs*. M1a, *p* = 0.020). However, no statistically significant associations were found between ESM-1 levels and age (*p* = 0.563), gender (*p* = 0.930), histological type (*p* = 0.756), PS (*p* = 0.515), tumor size (*p* = 0.218) and smoking (*p*=0.138).

**Table 2:** Levels of ESM-1 in MPE and association with the clinicopathological factors of NSCLC patients

Clinical variables	Number	ESM-1(ng/ml)	*P*-value
Age (years)			0.563
≥ 65	23	54.93 ± 8.12	
< 65	47	49.33 ± 5.46	
Gender			0.930
Male	40	50.82 ± 5.53	
Female	30	51.63 ± 7.61	
Histological type			0.756
Adenocarcinoma	53	51.97 ± 5.36	
Squamous cell carcinoma	17	48.6 7 ± 8.30	
Performance Status			0.515
0 to 1	35	48.20 ± 6.20	
2 to 4	35	54.14 ± 6.59	
Smoking status			0.138
Nonsmoker	36	44.65 ± 6.00	
Smoker	34	58.07 ± 6.65	
Tumor size (cm)			0.218
≤ 5	41	46.47 ± 5.17	
> 5	29	57.82 ± 8.01	
Distant metastasis			0.020^a^
Absent	31	39.18 ± 5.34	
Present	39	60.16 ± 6.49	

^a^Significant difference.

### Prognostic significance of pleural fluid ESM-1 for NSCLC patients

**Figure 3:**
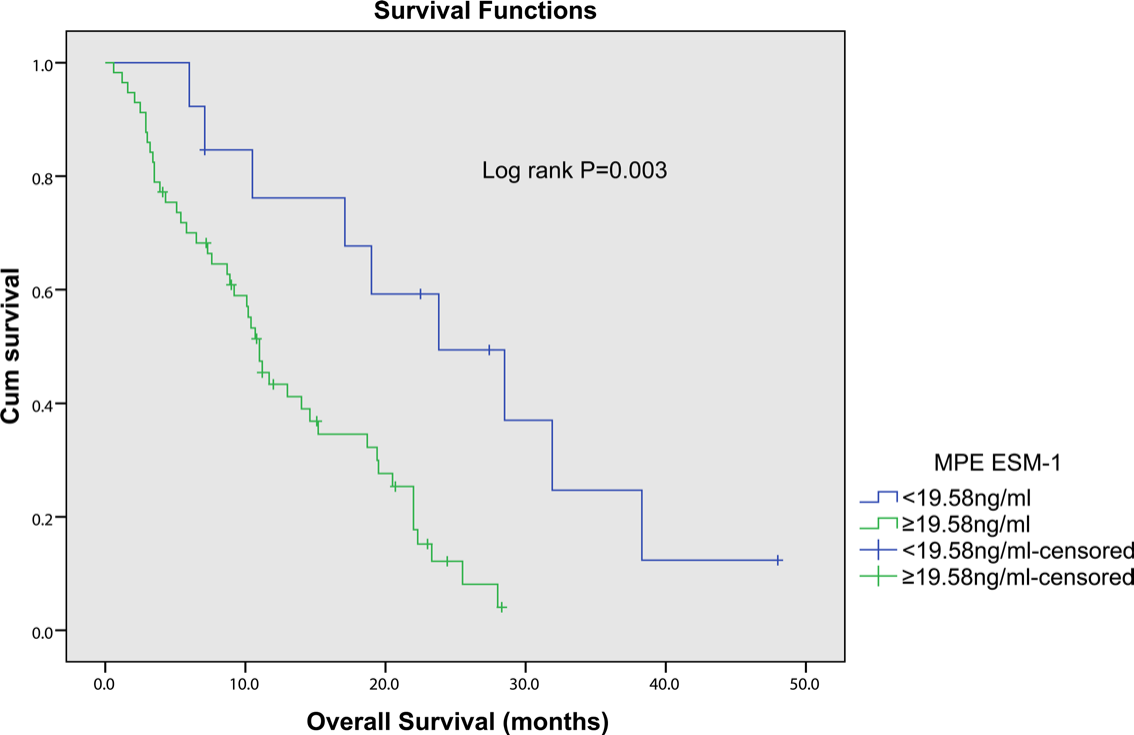
Kaplan-Meier survival curves for overall survival of NSCLC patients with different pleural fluid ESM-1 expression The NSCLC patients with high ESM-1 expression had a significantly worse outcome compared with the patients with low ESM-1 expression (*p* = 0.003).

NSCLC patients with pleural effusion were divided into low- and high-concentration of ESM-1 groups based on the optimal cutoff value (19.58ng/ml). Using Kaplan-Meier curves and log-rank test, the OS of NSCLC patients with high levels of pleural fluid ESM-1 was significantly shorter than those who with low levels of ESM-1 (Figure 3, *p* = 0.002). Univariate analysis (Table 3) showed that OS was associated with ESM-1(*p* = 0.002), PS (*p* = 0.025), tumor size (*p* = 0.045) and distant metastasis (*p* = 0.002), but not with age (*p* = 0.231), gender (*p* = 0.938), histological type (adenocarcinoma/squamous cell carcinoma, *p* = 0.460) and smoking index (*p* = 0.051). Moreover, to confirm the prognostic value of pleural fluid ESM-1, we performed multivariate analysis of prognostic factors using the Cox proportional hazards model. Multivariate Cox regression analysis revealed that pleural fluid ESM-1 level (HR 1.007, 95%CI, 1.000–1.014, *p* = 0.039), and distant metastasis status (HR 2.092, 95%CI, 1.017–4.302, *p* = 0.045) were independent prognostic markers for OS of NSCLC (Table 3). Thus, ESM-1 may represent a novel tumor biomarker for the prognosis and prognostic of patients with NSCLC.

**Table 3:** Univariate and multivariate Cox proportional hazards analysis of prognostic variables for overall survival in 70 NSCLC patients

Parameters	Categories	Univariate analysis HR (95% CI)	*P* value	Multivariate analysis HR (95% CI)	*P* value
Age	< 65 *vs* ≥ 65	0.712 (0.409–1.241)	0.231		
Gender	Male *vs* female	0.978 (0.552–1.731)	0.938		
Histological type	Ad *vs* Sq	0.792 (0.427–1.469)	0.460		
ECOG PS	2 to 4 *vs* 0 to 1	1.907 (1.083–3.358)	0.025^a^	1.450 (0.794–2.648)	0.245
Smoking status	Smoker *vs* Nonsmoker	0.835 (0.489–1.427)	0.510		
Tumor size (cm)	> 5 *vs* ≤ 5	1.799 (1.013–3.194)	0.045^a^	0.875 (0.420–1.822)	0.720
Distant metastasis	Present *vs* Absent	2.673 (1.392–4.444)	0.002^a^	2.092 (1.017–4.302)	0.045^a^
ESM-1 level	High *vs* Low	1.011 (1.004–1.017)	0.002^a^	1.007 (1.000–1.014)	0.039^a^

^a^Significant difference.

HR = hazard ratio; CI = confidence interval; Ad = adenocarcinoma; Sq = squamous cell carcinoma; ECOG PS = *Eastern Cooperative Oncology Group* Performance status.

## DISCUSSION

In the present study, we evaluated the levels of ESM-1 in NSCLC patients with MPE and BPE controls. Our results showed that the levels of pleural fluid ESM-1 were significantly increased in MPE than those in BPE controls. ROC analysis confirmed that pleural fluid ESM-1 level presented a relative high sensitivity and specificity for the diagnostic accuracy of MPE. Moreover, we found ESM-1 level in MPE was positively associated with distant metastasis. By multivariate and Cox regression analysis, we demonstrated that ESM-1 was an independent prognostic marker for OS of NSCLC. These findings imply that ESM-1 could be a promising non-invasive biomarker for NSCLC diagnosis and prognosis.

In the United States, there are approximately 220, 000 new diagnosis MPE patients each year in whom lung cancer is the most common cause [19, 20]. It is important to make an early diagnosis of MPE, because once NSCLC patients suffer from MPE, they should be classified into advanced stages and have limited life expectancies [21]. To detect malignant cells in pleural effusion or evidence of tumor invasion, pleural biopsy is considered to be the gold diagnostic standard for NSCLC patients with MPE. However, there are still many patients can't get a clear cytological diagnosis even by the examination of thoracoscopy. In addition, certain patients are not suitable for or willing to have invasive procedures. Therefore, it is occasionally difficult to make a definitive diagnosis of MPE for chest physicians in clinical practice.

ESM-1 is constituted of a mature polypeptide of 165 amino acids and a single dermatan sulfate chain covalently linked to the serine residue at position 137 [22]. It is synthesized and secreted by the vascular endothelial cells [22], and plays a vital role in the regulation of cell adhesion, inflammatory disorders, and tumor progression [23]. ESM-1 has been found highly over-expressed in some cancer tissues which could be a new biomarker for many cancers including lung cancer [9, 12, 15]. In gastric cancer, ESM-1 mRNA was significantly up-regulated in cancer tissues than that in adjacent normal tissues, and high ESM-1 level was associated with distant metastasis, Borrmann type IV and vascular invasion [12]. Additionally, serum ESM-1 was more sensitive than CEA for the diagnosis of gastric cancer [17]. In colorectal cancer patients, serum ESM-1 levels were significantly higher than those in healthy controls, and positively correlated with histological differentiation, depth of tumor invasion, TNM stage and lymph node metastasis [16]. Since either ESM-1 mRNA in colorectal cancer tissues or ESM-1 protein in serum were elevated in both early stage and advanced stage colorectal cancer [15], ESM-1 is recommended to be a serum biomaker for the early detection of colorectal cancer. Moreover, ESM-1 is also valuable for the diagnosis and follow-up of hepatocellular carcinoma [10] and renal clear cell carcinomas [11]. In the present study, we found that the level of ESM-1 is significantly higher in NSCLC-related MPE than that in BPE, and positively associated with distant metastasis of NSCLC. In normal tissues, ESM-1 mRNA is moderately detected in lung and kidney tissues, while no ESM-1 mRNA expression was detected in other endothelial cell-rich tissues, such as heart and placent [8]. However, it is highly over-expressed in some cancer tissues [23]. It is reported that tumor cell-derived factors such as vascular endothelial growth factor can stimulate vascular endothelial cells to increase the secretion of ESM-1 [24], which in turn bind hepatocyte growth factor/scatter factor through its dermatan sulfate chain and promote vascular endothelial cells proliferation and angiogenesis [25]. Considering the over-expression of ESM-1 in carcinoma endothelial cells and the vital roles of ESM-1 in cell adhesion, proliferation, and angiogenesis [26], it is reasonable that ESM-1 in pleural fluid may be a novel biomarker for pleural or distant metastasis of NSCLC.

Mounting evidence indicates serum ESM-1 could be an independent poor prognostic factor for gastric cancer [12, 17] and colorectal cancer [15, 16]. In NSCLC, Grigoriu BD et al. suggested that high level of serum ESM-1 predicted poor prognostic and short time to tumor progression [9]. However, serum ESM-1 level was not an independent prognostic factor for NSCLC. In our present study, ESM-1 levels in MPE were conversely correlated with NSCLC patients' OS. In other words, high pleural fluid ESM-1 levels appear to be a predictor of poor prognosis in NSCLC compared with low levels. Using Kaplan–Meier and multivariate Cox proportional hazards analyses, we further confirm that ESM-1 is an independent prognostic factor.

However, this study suffers from several limitations. First, the enrolled sample size was relatively small due to a retrospective study. A large controlled clinical trial will be carried out to confirm our conclusion. Second, although we examined the diagnostic and prognostic values of ESM-1 for MPE, this study did not evaluate the possible association between ESM-1 levels and susceptibility to platinum-based chemotherapy. Thus, further studies are warranted to elucidate whether ESM-1 could be a predictive factor for platinum-based therapy response.

In summary, pleural fluid ESM-1 concentration showed high diagnostic sensitivity, specificity, and accuracy to predict whether a pleural effusion is benign or malignant. Moreover, ESM-1 level in pleural effusion is valuable for prognosis evaluation and therapy selection for NSCLC patients with MPE.

## MATERIALS AND METHODS

### Patients and diagnostic criteria

This retrospective study has been approved by the Ethics Committee of Nanjing Chest Hospital and all patients signed informed consent. Consecutive patients with pleural effusion admitted to Nanjing Chest Hospital from July 2011 to April 2013 were enrolled into the study. Pleural effusion due to connective tissue diseases, cardiac failure, hypoproteinemia or kidney dysfunction were excluded. By cytologic or histologic diagnosis of pleural effusion, all the patients were divided into MPE or BPE group. The diagnostic criteria for MPE and BPE have been previously described [18]. Briefly, MPE caused by NSCLC was diagnosed on the basis of detecting malignant cells in pleural effusion and/or evidence of tumor invasion by pleural biopsy. Tuberculous pleural effusion was diagnosed with a high level of pleural fluid adenosine deaminase (> 40U/L), a pleural biopsy specimen showing typical epitheloid cell granuloma or presence of M.tuberculosis, and response to antituberculous therapy. Parapneumonic effusion was diagnosed with a glucose concentration > 3.3 mmol/L and PH > 7.2, and can be cured by anti-infection treatment. Of the total sample, 70 patients (58.33%) had MPE and 50 (41.67%) had BPE. The MPE group included 46 men and 24 women with a median age of 62 years (range 33–84 years). Patients with MPE received clinical follow-up and the overall survival (OS) time was measured from the time of diagnosis to the date of death or the last follow-up.

### Measurement of pleural effusion ESM-1

Fresh pleural fluid specimens obtained by therapeutic pleural aspiration, intercostal tube drainage, or endoscopic examination were taken from patients prior to therapy and centrifuged for 1,500 rpm × 10 minutes at −4°C, and then stored at −80°C immediately for further analysis. ESM-1 levels in pleural effusion were determination by ESM-1 ELISA kits (Uscn Life Science, Inc, Wuhan, Hubei Province, China). All these samples were detected according to manufacturer's instructions by investigators in our laboratory who were blind to patients' information.

### Statistical analysis

All statistical analyses were performed by SPSS software (SPSS 17.0, Chicago, IL, USA) and the GraphPad Prism 6 (GraphPad Software Inc., CA, USA); the data were characterized by mean ± SEM, a *p* value < 0.05 was considered as statistically significant. One-way ANOVA and/or unpaired Student's *t* test were used to detect the significant differences between groups. ROC curve was calculated to assess the diagnostic value of ESM-1. Survival curves were performed by Kaplan-Meier method and survival characteristics were compared by log-rank test. Univariable and multivariate Cox proportional hazards model were carried out to analysis the significance of different prognostic factors for OS, including age, gender, histological type, Eastern Cooperative Oncology Group Performance status (ECOG PS), Smoking index, distant metastasis and pleural fluid ESM-1 level.
